# Anesthetic Management of a Patient with Wolff-Parkinson-White Syndrome for Laparoscopic Cholecystectomy: A Case Report

**DOI:** 10.31729/jnma.5217

**Published:** 2020-09-30

**Authors:** Kiran Kumar KC, Sundar Hyoju, Pawankumar Raya

**Affiliations:** 1 Department of Anesthesiology and Intensive care Medicine, Nepal Police Hospital, Nepal

**Keywords:** *Accessory pathway*, *tachyarrhythmia*, *WPW syndrome*

## Abstract

Wolff-Parkinson-White syndrome, an electrophysiological disorder of heart caused by pre-excitation of an abnormal accessory pathway,can either be asymptomatic or may present with palpitation or exertional dyspnea. We report a case of an asymptomatic 45-year-old male with incidental finding of Wolff-Parkinson-White syndrome posted for laparoscopic cholecystectomy under general anesthesia. The anesthetic management of these patients is challenging as they are prone to develop life-threatening tachyarrhythmia. Taking all the necessary precautions to prevent tachyarrhythmia, balanced anesthesia, rigorous monitoring and preparedness with necessary drugs and equipment to treat any complications is the cornerstone for positive outcome.

## INTRODUCTION

Wolff-Parkinson-White (WPW) syndrome is caused by pre-excitation of an abnormal accessory pathway between atria and ventricle bypassing normal atria-ventricular route. The incidence is 0.9-3% of the general population.^1^ The diagnosis is done with history and electrocardiograph (ECG), which shows a shortened PR interval, delta waves and widened QRS complex.^2^ Patients can be asymptomatic or may present with palpitation or exertional dyspnea. Due to changes in conduction physiology by anesthetic drugs and cardiovascular alterations due to surgical stress response and effect of pneumoperitoneum, life-threatening tachyarrhythmia may be precipitated thus it is important to know about perioperative management of such condition. We are presenting this case of cholelithiasis with WPW syndrome posted for elective lap cholecystectomy.

## CASE REPORT

**Figure 1:**
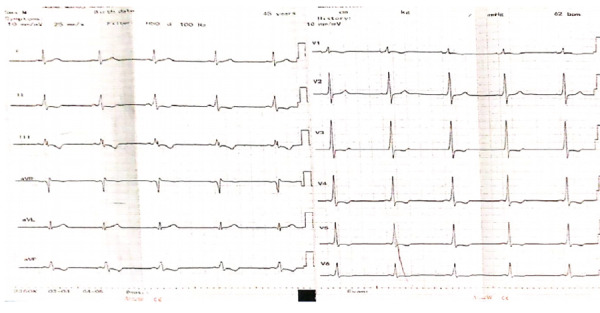
Pre-operative Resting ECG.

A 45-year-old male weighing 60 kg was planned for laparoscopic cholecystectomy for cholelithiasis. The patient denies of chest pain, palpitation and exertional dyspnea and his general and systemic examination were within normal limits with a regular heart rate of 70/min and BP of 110/74 mm of Hg. However, his 12 lead ECG showed shortened PR interval and presence of “delta” wave (Fig 1). ECHO showed LV diastolic dysfunction grade I and dilated LA with an ejection fraction of 65%. 24 hours Holter monitoring revealed sinus rhythm with a pre-excitation pattern, maximum heart rate of 146 and a minimum of 49 bpm a single ventricular event of arrhythmia which required no intervention.

The patient was counseled, possible complications explained and premedicated with tab alprazolam 0.5 mg in the night prior to surgery. The perioperative goal of anesthesia was to avoid any factor that causeda sympathetic surge.

In the operating room, ASA standard monitors were attached. Under lignocaine 2% infiltration, IV catheter, radial artery and central venous access were secured and invasive monitoring also done. A defibrillator along with adenosine, esmolol, lidocaine, procainamide, and amiodarone was kept ready. The patient was preoxygenated with 100% oxygen for 3 minutes followed by inj. Midazolam 2.5 mg, inj. fentanyl 2 mcg/kg, induction with titrating the dose of propofol, and rocuronium as muscle relaxant 10% lignocaine spray was used to anesthetize the airway. Intubation response was observed with a slight increase in heart rate and blood pressure with ECG showing prominent “delta waves”. Anesthesia was maintained with propofol infusion at the rate of 200-250 mcg/kg/min targeting the vitals throughout the surgery. CO2 insufflation pressure was limited to 12 while creating pneumoperitoneum and Et CO2 between 35-40 mm of Hg. Further patient positioning also had no effect on hemodynamics. The case proceeded uneventfully and the patient was extubated in a deep plane after reversal following spontaneous breathing. Propofol infusion was stopped following extubation. Extubation reflexes were not observed. The patient was successfully managed and comfortably shifted to the postoperative ward.

## DISCUSSION

Many cases of asymptomatic WPW syndrome are described, but such patients are always at a risk of developing reentrant tachyarrhythmia, even ventricular fibrillation, and sudden death during the perioperative period.^3^ The accessory conduction pathway between the atria and the ventricle allows electrical conduction at a site other than the AV node that does not share the rate slowing property of the AV node. Anesthetic drugs and techniques further tend to change the physiology of AV conduction.^4^

The perioperative anesthetic goal is to avoid sympathetic surge caused by pain, anxiety, the stress response of intubation and extubation reflex, the lighter plane of anesthesia and hypovolemia. Propofol is preferred as it has no effect on the refractory period of the accessory pathway. Sumhiko Seki, et al^5^ reported about the normalization of PR interval and wide QRS complexes with propofol infusion, but no such changes were observed in this case. Isoflurane and sevoflurane also have no effect on AV node conduction and provide the optimal hemodynamic status. Fentanyl provides adequate hemodynamic stability. Cardiostable muscle relaxants like Vecuronium and rocuronium are preferred over pancuronium and atracurium.^6^

Supraventricular Tachycardia during anesthesia should be managed by cardioversion or drugs depending on hemodynamic stability. Vagal maneuvers can be tried. Lidocaine, Adenosine, Class-I anti-arrythmics, beta-blockers are preferred drugs. Anti-arrhythmic drugs like Digoxin and Verapamil should be avoided as it could enhance anterograde conductions through an accessory pathway.^7^

To conclude, patients with WPW syndrome undergoing surgery can be managed safely under general anesthesia by taking adequate precautions to prevent and manage arrhythmias.

## Consent:

**JNMA Case Report Consent Form** was signed by the patient and the original article is attached withthe patient's chart.

## Conflict of Interest

**None.**

## References

[1] Rosner MH, Brady WJ, Kefer MP, Martin ML (1999). Electrocardiography in the patient with the Wolff-Parkinson-White syndrome: diagnostic and initial therapeutic issues. Am J Emerg Med.

[2] Mark DG, Brady WJ, Pines JM (2009). Preexcitation syndromes: diagnostic consideration in the ED. Am J Emerg Med.

[3] Klein GJ, Bashore TM, Sellers TD, Pritchett EL, Smith WM, Gallagher JJ (1979). Ventricular fibrillation in the Wolff-Parkinson-White syndrome. N Engl J Med.

[4] Sahu S, Karna ST, Karna A, Lata I, Kapoor D (2011). Anaesthetic management of Wolff-Parkinson-White syndrome for hysterectomy. Indian J Anaesth.

[5] Seki S, Ichimiya T, Tsuchida H, Namiki A (1999). A case of normalization of Wolff-Parkinson-White syndrome conduction during propofol anesthesia. Anesthesiology.

[6] Gupta A, Sharma J, Banerjee N, Sood R (2013). Anesthetic management in a patient with Wolff-Parkinson-White syndrome for laparoscopic cholecystectomy. Anesth Essays Res.

[7] Kabade SD, Sheikh S, Periyadka B (2011). Anaesthetic management of a case of Wolff-Parkinson-White syndrome. Indian J Anaesth.

